# Multicenter clinical application of regulatory dual-channel digestive tract reconstruction with pylorus-preserving approach in advanced gastric cancer

**DOI:** 10.3389/fonc.2026.1673727

**Published:** 2026-05-01

**Authors:** Hangyu Zhou, Ranmiao Li, Jin Chen, Rui Ming, Yu Yang, Guomin Zhou, Huaiwu Jiang

**Affiliations:** Department of General Surgery, Mianyang 404 Hospital, Mianyang, Sichuan, China

**Keywords:** dual-channel digestive tract reconstruction, gastric cancer, multicentric (MC), pylorus, quality of life

## Abstract

**Background:**

To evaluate the clinical significance of regulatory dual-channel digestive tract reconstruction with a pylorus-preserving approach in advanced gastric cancer through a multicenter study.

**Methods:**

A total of 137 gastric cancer patients who underwent radical gastrectomy at three hospitals in Mianyang, China, between March 2018 and March 2023 were included. Patients were divided into a study group (n=68) undergoing regulatory dual-channel reconstruction with pylorus preservation and a control group (n=69) receiving total gastrectomy with Roux-en-Y reconstruction. Operative time, intraoperative blood loss, postoperative complications, and quality of life were compared.

**Results:**

No significant differences were found between the groups regarding demographic and clinical characteristics, operative time, intraoperative blood loss, gastrointestinal function recovery, or hospital stay (P>0.05). Postoperative complications, including pulmonary infections, anastomotic leakage, gastrointestinal bleeding, and reflux esophagitis, also showed no significant differences (P>0.05). However, dumping syndrome was significantly less frequent in the study group (P<0.05). Both groups showed improvement in functional status and overall health from 1 to 24 months postoperatively. The study group had significantly higher physical function scores at 24 months (P<0.05), along with better emotional function and overall health scores from 6 to 24 months (P<0.05). Additionally, diarrhea scores were significantly lower in the study group at 1 and 6 months (P<0.05), while other functional and symptom domains showed no differences (P>0.05).

**Conclusion:**

Regulatory dual-channel digestive tract reconstruction with pylorus preservation is a safe and effective approach that improves postoperative quality of life in patients with advanced gastric cancer.

**Synopsis:**

Pylorus-preserving dual-channel reconstruction in advanced gastric cancer improves quality of life, reduces dumping syndrome, and shows comparable safety to total gastrectomy with Roux-en-Y.

## Introduction

One of the most common malignant tumors of the digestive tract is gastric cancer. It accounts for 5.6% of all cancer cases globally, according to the Global Cancer Statistics 2022 report (1), ranking fifth among 36 types of cancer. The latest national cancer report released by the National Cancer Center in 2018 in China demonstrated that the incidence rate of gastric cancer attained 30.00 per 100, 000, second after lung cancer. The gastric cancer incidence increases with age, and the incidence and mortality rates of gastric cancer are anticipated to increase with the aging population in China. Patients with gastric cancer suffer from physical pain owing to the disease, a poor quality of life (QOL), and a significant economic burden imposed on their families and society (2, 3). Studies indicate that the global incidence of gastric cancer is projected to continue its downward trajectory, a trend that is evident not only in historically high-incidence countries such as Japan and Belarus but also in low-incidence settings like Australia and the United Kingdom. Specifically, Japan’s age-standardized incidence rate (ASR) is anticipated to decline from 36 in 2010 to 30 in 2035, whereas Belarus is expected to see a reduction from 19 to 16 over the same interval; similarly, Australia and the United Kingdom are forecasted to experience decreases from 5.1 and 5.2 to 4.6 and 4.7, respectively (Arnold et al., 2020) (4). Although high resection rates and favorable 5-year survival rates have been reported in early-stage gastric cancer (5), the treatment outcomes for advanced gastric cancer remain unacceptable, with the surgical intervention being the primary therapeutic approach (6). Surgical treatment comprises R0 resection of the tumor, D2 lymphadenectomy, and reconstruction of the digestive tract. Although there are numerous modalities for digestive tract reconstruction following gastrectomy, a consensus has been achieved on R0 resection and D2 lymphadenectomy. However, to date, no single reconstruction technique has been recognized as ideal for simultaneously enhancing the postoperative QOL and improving long-term outcomes. Currently, there is no globally accepted standard for the optimal reconstruction technique, highlighting the requirement for innovative research on digestive tract reconstruction techniques following gastrectomy. Therefore, this study explores the clinical application of regulatory dual-channel digestive tract reconstruction with pylorus preservation in patients with advanced gastric cancer.

## Materials and methods

### General data

This study selected patients diagnosed with gastric cancer and undergoing radical gastrectomy at the three hospitals in Mianyang City (Mianyang 404 Hospital, Mianyang Third People’s Hospital, and Mianyang Traditional Chinese Medicine Hospital) between March 2018 and March 2023. The inclusion criteria were as follows: pathologically confirmed gastric cancer, locally advanced proximal gastric cancer(TNM stage T2 or higher) that was planned for radical gastrectomy, patients aged ≤ 75 years regardless of sex, Karnofsky Performance Status (KPS) score≥80, patients who participated voluntarily with signed informed consent, and the patients with the ability to independently complete the QOL questionnaire. The exclusion criteria were as follows: patients with early-stage gastric cancer; those with distant metastasis; patients with KPS<80 or poor general condition making surgery intolerable; those with a history of bowel surgery, cholecystectomy, or pancreatic surgery; those with functional dyspepsia; those with severe cardiac, pulmonary, hepatic, or renal dysfunction; those with other conditions affecting QOL; or those who were unable to comprehend or refused to sign informed consent. Patients were excluded if they did not fulfill the inclusion criteria, failed to complete the planned surgery, or had missing primary outcome measures or incomplete data. Withdrawal criteria were as follows: when the patients requested to withdraw, those with severe conditions unrelated to digestive tract reconstruction, those with poor compliance or inability to follow the study protocol, or those who developed other severe illnesses preventing follow-up observation. A total of 137 patients were finally included and, using a random number table (First, patients were numbered (01-137), and then the starting point and reading direction were randomly determined on the random number table. Patients were assigned to each group based on the parity of the extracted numbers.), randomly assigned into two groups as follows: 68 cases in the study group and 69 in the control group who underwent regulatory dual-channel reconstruction with pylorus preservation and total gastrectomy with Roux-en-Y reconstruction, respectively. The ethics committee of the hospital approved this study. All research team members received training before the initiation of the study with the implementation of the quality control measures. The participating hospitals were competent to perform both regulatory dual-channel digestive tract reconstruction and Roux-en-Y reconstruction for gastric cancer. In addition, the hospitals were equipped to conduct preoperative and postoperative follow-up assessments, including hematological tests, evaluation of nutritional status, and QOL surveys.The entire research process strictly adhered to ethical principles. The study protocol was approved by the Biomedical Ethics Committee of Sichuan Mianyang 404 Hospital (Approval No. 028, 2019). All study participants provided written informed consent with full awareness prior to enrollment.

### Methods

#### Regulatory dual-channel digestive tract reconstruction

**Figure 1:**
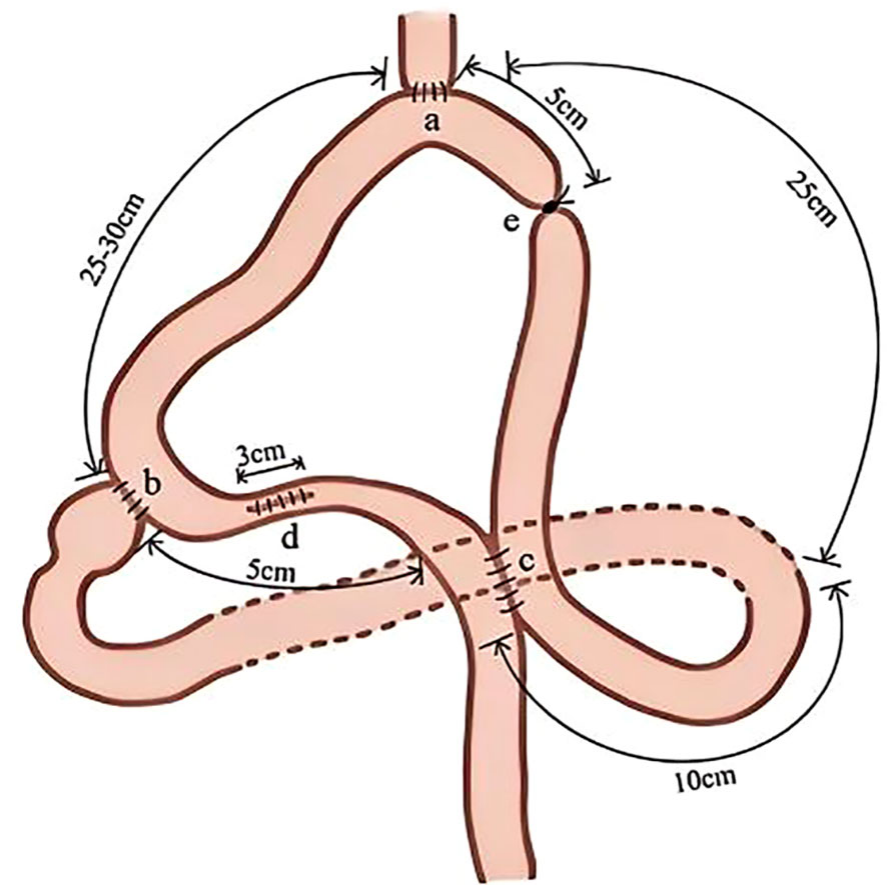
Schematic representation of the modified double-channel gastrointestinal reconstruction.

**Figure 2:**
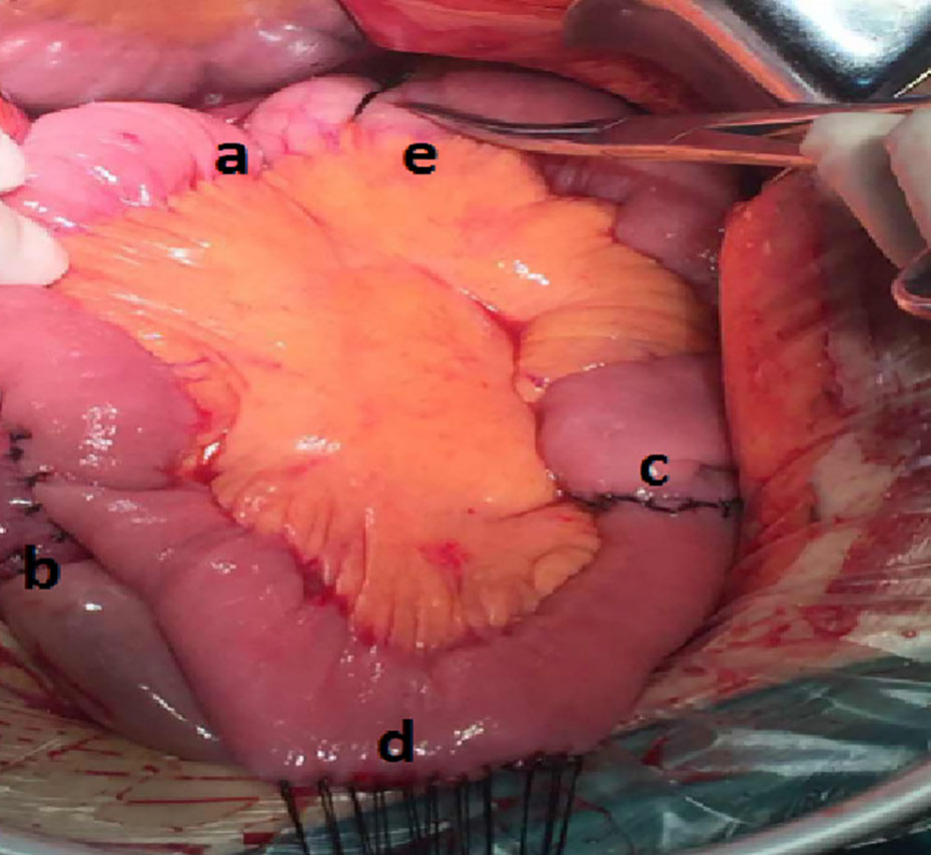
Pylorus-preserving regulated double-channel gastrointestinal reconstruction surgery: **(a)** Esophagojejunostomy; **(b)** Pylorojejunostomy; **(c)** Braun anastomosis; **(d)** Partially narrowed channel; and **(e)** Moderate ligation with thick sutures.

The jejunum was first elevated after proximal gastrectomy to approximately 25 cm distal to the ligament of Treitz. An end-to-side anastomosis was performed between the jejunum and esophagus. Subsequently, the jejunum was anastomosed end-to-side with the residual stomach at 35 cm distal to the esophagojejunostomy. Following this, a Braun anastomosis was created between the jejunum located approximately 5 cm distal to the gastrojejunostomy and the distal jejunum approximately 10 cm from the ligament of Treitz. A longitudinal incision was placed between the gastrojejunostomy and Braun anastomosis in the intestinal segment. A stapler was subsequently introduced through this incision to complete the anastomosis, followed by suturing of the incision, allowing the intestinal lumen to naturally constrict. The body of the stapler was inserted through this site for all anastomoses, with the anvil positioned at the esophageal stump, residual stomach, and proximal jejunum. The intestinal opening at this site was routinely sutured following the completion of all anastomoses, and the anastomotic site was embedded using a 3–0 silk seromuscular suture, ensuring that the diameter of the intestine was reduced to approximately one-third to one-half of the adjacent normal intestinal lumen. Thus, this approach aimed to direct intestinal contents through the duodenal passage to the maximum extent, while alleviating the pressure at the anastomotic site in the duodenal channel. Finally, to prevent postoperative recanalization of the afferent loop, the afferent loop of the jejunum, situated 5 cm from the esophagojejunostomy and the Braun anastomosis, was moderately ligated using a large round needle 1–0 double silk sutures at the mesenteric attachment site. The ligature traversed through the mesentery without damaging the intestinal wall, averting migration and thus eliminating the need for the seromuscular embedding of the small intestine (**Figures 1** and **2**).

**Figure 3:**
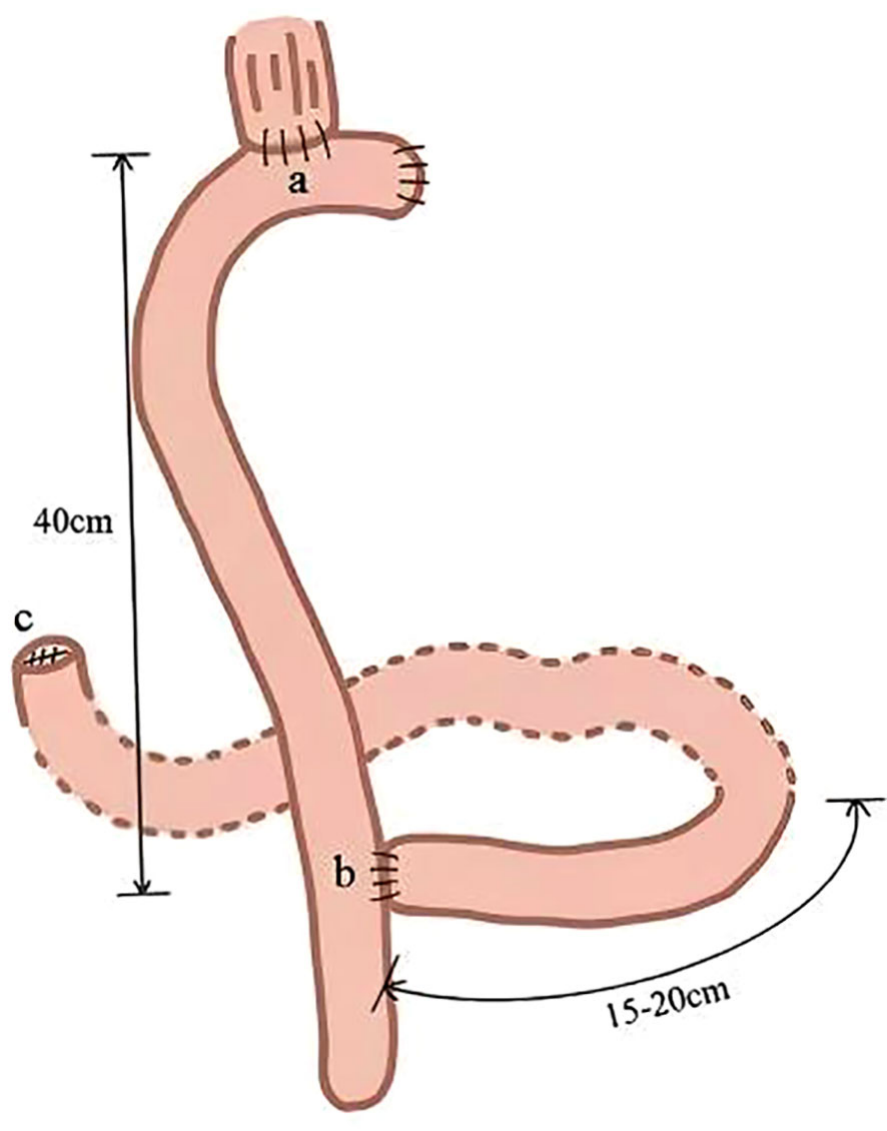
Schematic diagram of the Roux-en-Y reconstruction.

**Roux-en-Y Reconstruction:** The jejunum was transected approximately 15–20 cm distal to the ligament of Treitz following total gastrectomy. Following this, an end-to-side anastomosis was performed between the distal jejunum and the lower end of the esophagus after raising it anterior to the transverse colon. The proximal jejunum was anastomosed end-to-side with the distal jejunum at a location 40 cm below the esophagojejunostomy. Finally, the proximal end of the duodenum was approximated (**Figure 3**).

#### Comparison of the two digestive tract reconstruction methods

The differences between the two digestive tract reconstruction techniques were compared in terms of operative time, intraoperative blood loss, postoperative complications, and QOL.

#### Observation indicators

Surgical-related data, including operative time, intraoperative blood loss, postoperative anal exhaust time, time to initiate liquid diet, length of hospital stay, surgical complications, and QOL analysis, were collected and compared between the study and control groups.

#### Statistical analysis

Quantitative data following normal distribution were presented as mean ± standard deviation (x̅ ± s), while frequency (n) and percentage (%) were used to express categorical data. SPSS 20.0 software was used to enter and process all data. Based on the type of data and statistical methods applicable to each group, statistical analysis was performed using a chi-square test, analysis of variance, and t-test. The significance level was set at α=0.05, with statistical significance set at P<0.05.

## Results

### Comparison of clinical characteristics between the two groups

In a comparison of the clinical characteristics between the two digestive tract reconstruction methods, no significant differences were observed in terms of age, sex, BMI, tumor staging, histological classification, degree of differentiation, and the number of lymph nodes dissected (P>0.05). **Table 1** presents these results.

**Table 1:** Comparison of clinical characteristics between the two groups.

Clinical parameters	Study group (n=68)	Control group (n=69)	x²/t	P
Age (years)				
<50	12	9	0.56	>0.05
≥50	56	60		
Gender				
Male	41	39	0.20	>0.05
Female	27	30		
BMI(kg/m²)	18.7 ± 2.1	19.3 ± 2.8	1.420	>0.05
Tumor stage				
Stage I	5	7	1.26	>0.05
Stage II	27	33		
Stage III	34	29		
Histological type				
Tubular Adenocarcinoma	32	27	1.73	>0.05
Papillary Adenocarcinoma	9	11		
Mucinous Adenocarcinoma	20	26		
Signet Ring Cell Carcinoma	7	5		
Degree of differentiation				
Well-differentiated	17	15	0.40	>0.05
Moderately-differentiated	29	33		
Poorly-differentiated	22	21		
Number of lymph nodes dissected				
≥15	61	59	0.56	>0.05
<15	7	10		

### Comparison of perioperative parameters between the two groups

No statistically significant differences were observed between the groups in terms of surgical time, intraoperative blood loss, postoperative gastrointestinal function recovery time, or length of hospital stay (P>0.05) when the two digestive tract reconstruction techniques were compared. No significant differences in pulmonary infection, anastomotic leakage, gastrointestinal bleeding, wound infection, abdominal infection, intestinal obstruction, and reflux esophagitis (P>0.05) were observed in the statistical analysis of postoperative complications. However, a statistically significant difference was noted in the occurrence of dumping syndrome postoperatively (P<0.05) (**Table 2**).

**Table 2:** Comparison of perioperative parameters between the two groups.

Clinical parameters	Study group (n=68)	Control group (n=69)	t/x²	P
Surgical Time (min)	187.91 ± 8.386	185.35 ± 7.66	1.87	0.060
Intraoperative Blood Loss (ml)	120.44 ± 38.53	124.49 ± 36.92	0.63	0.531
Time to First Flatus (days)	4.81 ± 0.68	4.59 ± 0.63	1.93	0.056
Time to Start Liquid Diet (days)	5.96 ± 0.66	5.77 ± 0.60	1.75	0.082
Length of Hospital Stay (days)	11.93 ± 1.73	11.48 ± 0.95	1.88	0.062
Postoperative complications (n)				
Pulmonary Infection	9/68	7/69	0.32	0.18
Anastomotic Leakage	3/68	2/69	0.00	0.31
Gastrointestinal Bleeding	2/68	1/69	0.00	0.37
Wound Infection	4/68	5/69	0.00	0.26
Abdominal Infection	6/68	5/69	0.10	0.23
Intestinal Obstruction	3/68	4/69	0.00	0.28
Dumping Syndrome	0/68	4/69	2.27	0.03*
Reflux Esophagitis	2/68	3/69	0.00	0.32

*The difference in dumping syndrome between the two groups was statistically significant (P<0.05).

### Comparison of quality of life between the two groups

The QOL in both groups was assessed using the European Organization for Research and Treatment of Cancer Quality of Life Questionnaire-Core 30 (EORTC QLQ-C30). The EORTC QLQ-C30 comprises 30 items, including five functional scales (physical, role, cognitive, emotional, and social functions), three symptom scales (fatigue, nausea and vomiting, and pain), six single-item measurements, and one overall health status (**Table 3**).

**Table 3:** EORTC QLQ-C30 cancer patient quality of life questionnaire scores.

Clinical parameter	Group	1 month post-op	6 month post-op	12 month post-op	24 month post-op
Physical Function	Study Group	61.86 ± 10.23	66.67 ± 7.81	73.04 ± 5.45	79.71 ± 4.22
	Control Group	62.22 ± 10.01	67.63 ± 6.69	72.66 ± 5.96	77.87 ± 5.66
	P	0.835	0.438	0.695	0.034*
Role Function	Study Group	54.90 ± 10.79	58.83 ± 10.17	73.04 ± 5.45	74.27 ± 8.36
	Control Group	55.07 ± 11.89	59.18 ± 8.83	72.66 ± 5.96	72.95 ± 8.13
	P	0.930	0.827	0.695	0.351
Social Function	Study Group	44.61 ± 12.02	60.54 ± 8.59	72.06 ± 7.85	76.96 ± 9.56
	Control Group	47.82 ± 11.04	61.35 ± 8.81	74.15 ± 7.34	75.60 ± 9.72
	P	0.105	0.585	0.133	0.412
Cognitive Function	Study Group	43.38 ± 11.21	58.33 ± 10.58	73.53 ± 10.47	80.14 ± 9.22
	Control Group	44.44 ± 10.95	57.25 ± 12.61	74.15 ± 10.12	80.43 ± 9.46
	P	0.576	0.586	0.723	0.857
Emotional Function	Study Group	58.09 ± 7.05	64.71 ± 5.59	68.99 ± 4.95	79.16 ± 4.19
	Control Group	57.73 ± 7.47	61.23 ± 6.52	66.91 ± 5.53	76.81 ± 5.51
	P	0.773	0.001*	0.022*	0.006*
Overall Health Status	Study Group	46.94 ± 5.36	62.13 ± 6.35	67.03 ± 6.18	74.02 ± 7.68
	Control Group	47.83 ± 5.27	59.06 ± 5.30	64.25 ± 5.73	71.01 ± 6.33
	P	0.330	0.003*	0.007*	0.014*
Fatigue	Study Group	66.83 ± 7.31	58.99 ± 6.72	48.53 ± 8.57	45.75 ± 7.80
	Control Group	67.64 ± 7.06	59.91 ± 7.43	49.60 ± 9.45	47.18 ± 7.48
	P	0.514	0.451	0.490	0.275
Nausea & Vomiting	Study Group	43.38 ± 10.44	27.45 ± 8.02	26.22 ± 8.30	22.79 ± 9.06
	Control Group	46.13 ± 11.13	28.02 ± 8.80	25.85 ± 8.35	24.88 ± 8.39
	P	0.138	0.694	0.790	0.165
Pain	Study Group	71.57 ± 11.54	63.23 ± 12.06	39.95 ± 11.20	26.22 ± 9.24
	Control Group	72.46 ± 11.72	67.15 ± 12.11	40.10 ± 11.89	26.32 ± 8.76
	P	0.653	0.060	0.941	0.947
Dyspnea	Study Group	53.43 ± 16.43	51.47 ± 16.73	49.02 ± 16.76	47.05 ± 16.53
	Control Group	56.52 ± 15.45	54.59 ± 16.14	49.76 ± 16.79	47.34 ± 16.58
	P	0.259	0.269	0.797	0.920
Insomnia	Study Group	66.67 ± 14.11	47.06 ± 16.53	45.59 ± 16.19	43.13 ± 15.30
	Control Group	70.05 ± 16.31	49.76 ± 16.79	46.38 ± 16.39	44.93 ± 15.99
	P	0.197	0.345	0.777	0.504
Loss of Appetite	Study Group	50.49 ± 16.78	49.50 ± 16.79	45.59 ± 16.19	43.14 ± 15.30
	Control Group	52.17 ± 16.64	51.21 ± 16.75	46.38 ± 16.39	43.96 ± 15.65
	P	0.556	0.554	0.777	0.756
Constipation	Study Group	59.81 ± 13.58	58.83 ± 14.25	52.94 ± 16.53	50.98 ± 16.76
	Control Group	60.87 ± 12.73	59.91 ± 13.51	54.59 ± 16.14	53.14 ± 16.49
	P	0.636	0.650	0.556	0.448
Diarrhea	Study Group	44.61 ± 15.89	45.59 ± 16.19	46.08 ± 16.32	46.56 ± 16.43
	Control Group	51.69 ± 16.71	52.66 ± 16.58	48.79 ± 16.75	47.83 ± 16.65
	P	0.012*	0.013*	0.339	0.657
Economic Difficulty	Study Group	72.06 ± 15.89	68.63 ± 23.66	63.24 ± 18.34	62.74 ± 18.69
	Control Group	71.98 ± 15.78	71.01 ± 22.80	63.77 ± 17.84	65.70 ± 17.12
	P	0.977	0.549	0.863	0.336

*Statistical significance between groups: P < 0.05.

A linear transformation was applied using a range normalization method to allow comparison across different domains, converting raw scores into standardized scores within a range of 0 to 100. In this scoring system, higher scores in the functional and overall health status domains indicate a better QOL, while higher scores in the symptom domains suggest more pronounced symptoms, thus suggesting a worse QOL (7, 8).

In the functional and overall health status domains, the scores of patients who underwent the double reconstruction surgery demonstrated an increasing trend from 1 to 24 months post-surgery. In the symptom domains, the scores of both groups gradually decreased over time. In the comparison of physical function scores, statistical analysis revealed that the study group had a significantly higher score than the control group at 24 months post-surgery (P<0.05). In comparing emotional function and overall health status scores, the study group scored significantly higher than the control group from 6 to 24 months post-surgery (P<0.05). In the symptom domain, the study group had significantly lower diarrhea scores than the control group at 1 and 6 months post-surgery (P<0.05). No significant differences were observed between the two groups in other functional or symptom domains (P>0.05).

## Discussion

The selection of digestive tract reconstruction modalities after radical gastric cancer resection has been debatable. The aim is to achieve the maximum preservation of gastrointestinal physiological functions, ensuring a high QOL for patients following surgery. The critical factors affecting the QOL of patients are post-reconstruction complications, such as gastroesophageal reflux, dumping syndrome, emptying disorders, and indigestion (9, 10). Currently, no evident evaluation system exists for the reconstruction of the digestive tract after gastric cancer surgery. Both the research and control groups showed a certain degree of surgical safety and efficacy, with no significant differences in surgical time, intraoperative blood loss, and gastrointestinal recovery time. This study discovered that gastric antrum preservation using a modified double-channel digestive tract reconstruction was comparable to the traditional Roux-en-Y anastomosis with regard to safety and feasibility, without an increase in surgical time, blood loss, or postoperative complications.

In the early postoperative feedback on dumping syndrome, the research group demonstrated a slight advantage, possibly because of the preservation of the gastric antrum and duodenal pathway in the procedure. The modified double-channel reconstruction maintains both the gastric and duodenal pathways, preserving the gastric antrum, while ensuring the continuity of the neuro-muscular structure of the intestinal tract. In general, food transport is facilitated through two channels, with suturing causing the narrowing of one channel. Since this channel is constricted, the food is redirected primarily through the physiological pathway of the duodenum because of the changes in gastrointestinal pressure and compliance. Liquids and bile reflux can be diverted through the narrowed intestinal segment, referred to as the “regulatory channel.” This approach preserves a functional anatomical reconstruction, consistent with normal physiological conditions and theoretically minimizes complications such as reflux, blind loops, and food retention. The preservation of the antrum is crucial. The pyloric sphincter acts like a “valve”, opening and closing regularly to effectively prevent the rapid flow of stomach contents into the small intestine. This prevents the sudden entry of food into the jejunum, which causes a high osmotic load. And this high osmotic load is the core factor that triggers the dumping syndrome (especially the early dumping). In the QOL score analysis, the research group experienced fewer episodes of diarrhea at 1 and 6 months postoperatively than that in the control group, with a statistically significant difference. The preservation of the gastric antrum also promotes the secretion of gastrin, secretin, cholecystokinin, and other digestive hormones, further enhancing the secretion of digestive fluids. This can increase the digestion of food and absorption of nutrients, particularly the absorption of vitamins such as D and B12, which are primarily absorbed in the duodenum. The absorption of these vitamins is supported by preserving the duodenal pathway (11, 12). In patients after gastric cancer surgery, clinical symptoms, such as pain, dyspnea, and insomnia, gradually disappear over time. The research group consistently scored higher than the control group for physical function, with emotional well-being and overall health status demonstrating improvement with time, and the differences being statistically significant. However, this difference became more apparent 6 months post-surgery and remained persistent for a longer period. Therefore, a slight long-term advantage is noted in the modified double-channel reconstruction with gastric antrum preservation in terms of QOL, potentially related to the changes in gastrointestinal microecology post-gastric surgery. This could influence the expression of certain biomarkers, such as neurotransmitters, thereby causing an impact on the emotional state, physical function, and overall health of the patients.

Thus, the association between the expression of gastric cancer-related molecular biomarkers and digestive tract reconstruction should be further explored. The goal of gastric cancer surgery is primarily on functional reconstruction along with anatomical reconstruction. The ultimate aim of gastrointestinal surgeons is to pursue high QOL for patients. Maintaining gastrointestinal microecology plays a critical role in postoperative gastrointestinal function and long-term QOL. Although this is a multicenter clinical comparative study, the number of clinical cases remains limited, and further data enrichment and analysis are necessary. Integrating clinical objective indicators with QOL assessments is essential to establish a comprehensive QOL evaluation system. This will provide scientific evidence for selecting the most appropriate digestive tract reconstruction method for gastric cancer surgery and improving surgical outcomes.

## Conclusions

In patients with advanced gastric cancer, regulatory dual-channel digestive tract reconstruction using a pylorus-preserving approach exhibits safety, efficacy, and improved quality of life.

## Data Availability

The original contributions presented in the study are included in the article/supplementary material. Further inquiries can be directed to the corresponding author.
